# Supplementary material for: The Systems Biology Research Tool: evolvable open-source software
Source: BMC Syst Biol. 2008 Jun 29;2:55. doi: 10.1186/1752-0509-2-55 (PMC2446383; doi:10.1186/1752-0509-2-55)
Supplement: Additional file 1 — SBRT Archive. An archive of the current version of the Systems Biology Research Tool. [file 1752-0509-2-55-S1.zip › sbrt-1.4.0/doc/developers_guide/api/index-files/index-19.html]

 
 
 
 
 
 
S-Index
 


 

 
function windowTitle()
{
    parent.document.title="S-Index";
}
 
 
 

 

 


 
   
  
 
 
 
   
 
   
          Overview     
        Package   
        Class   
        Use   
          Tree     
          Deprecated     
       Index    
          Help     
   
 
 
  
 
 
 

 
  
   PREV LETTER   
   NEXT LETTER    
  
    FRAMES     
   NO FRAMES     
  
    All Classes  ');
  }
  //-->
 
 
    All Classes  
 


  
 
 
  
 

 A   B   C   D   E   F   G   H   I   J   K   L   M   N   O   P   Q   R   S   T   U   V   W   X   Y   Z   
    
 S  
 
   SAFETY_LEVEL   - 
Static variable in class sbrt.shell.mng.fba. FbaOptManager 
 The keyword used to denote the safety level.
   SAMPLE_A_FILE   - 
Static variable in class sbrt.shell.mng.stat. MannWhitneyUManager 
 The keyword used to denote the name of the file containing sample A.
   SAMPLE_B_FILE   - 
Static variable in class sbrt.shell.mng.stat. MannWhitneyUManager 
 The keyword used to denote the name of the file containing sample B.
   SBML_INPUT_FILE   - 
Static variable in class sbrt.shell.mng.fba. BiggSbmlReaderManager 
 The keyword used to denote the name of the SBML input file.
   SBML_INPUT_FILE   - 
Static variable in class sbrt.shell.mng.fba. BiggSbmlTranslaterManager 
 The keyword used to denote the name of the SBML input file.
   SBML_INPUT_FILE   - 
Static variable in class sbrt.shell.mng.fba. PalssonSbmlReaderManager 
 The keyword used to denote the name of the SBML input file.
   SBML_INPUT_FILE   - 
Static variable in class sbrt.shell.mng.fba. PalssonSbmlTranslaterManager 
 The keyword used to denote the name of the SBML input file.
   SBML_OUTPUT_FILE_NAME   - 
Static variable in class sbrt.shell.mng.fba. SbmlWriterManager 
 The keyword used to denote the name of the SMBL output file.
   SbmlWriter   - Class in  sbrt.shell.proc.fba  This class is used to write fluxomes in SBML format.   SbmlWriter()   - 
Constructor for class sbrt.shell.proc.fba. SbmlWriter 
  
   SbmlWriterManager   - Class in  sbrt.shell.mng.fba  This class is used to manage the translation of Systems Biology Markup Language (SBML)
 files into Systems Biology Research Tool (SBRT) Fluxome Files.   SbmlWriterManager()   - 
Constructor for class sbrt.shell.mng.fba. SbmlWriterManager 
  
   sbrt   - package sbrt Provides the class used to run the Systems Biology Research Tool.   Sbrt   - Class in  sbrt  This class is used to execute the Systems Biology Research Tool as an
 application.   Sbrt()   - 
Constructor for class sbrt. Sbrt 
  
   sbrt.ext   - package sbrt.ext Provides classes that allow the Systems Biology Research Tool to
encapsulate external software.   sbrt.ext.cplex   - package sbrt.ext.cplex Provides a class that allows communication with CPLEX.   sbrt.ext.glpk   - package sbrt.ext.glpk Provides a class that allows communication with GLPK.   sbrt.ext.mma   - package sbrt.ext.mma Provides classes that allow communication with Mathematica.   sbrt.ext.mma.algebra   - package sbrt.ext.mma.algebra Provides classes that allow communication with Mathematica for the
purposes of algebra.   sbrt.ext.R   - package sbrt.ext.R Provides classes that allow communication with R.   sbrt.ext.R.stat   - package sbrt.ext.R.stat Provides classes that allow communication with R for the purposes of
statistics.   sbrt.kernel   - package sbrt.kernel Provides interfaces to define processes.   sbrt.kernel.fba   - package sbrt.kernel.fba Provides classes and interfaces for creating objects relevant to 
Flux Balance Analysis.   sbrt.kernel.fba.analysis   - package sbrt.kernel.fba.analysis Provides classes and interfaces for analyzing Flux Balance Analysis data.   sbrt.kernel.fba.comp   - package sbrt.kernel.fba.comp Provides classes and interfaces for performing Flux Balance Analysis computations.   sbrt.kernel.fba.proc   - package sbrt.kernel.fba.proc Provides classes and interfaces for executing Flux Balance Analysis processes.   sbrt.kernel.io   - package sbrt.kernel.io Provides classes and interfaces used to perform kernel-level I/O.   sbrt.kernel.math   - package sbrt.kernel.math Provides classes and interfaces for creating objects relevant to mathematics.   sbrt.kernel.math.algebra   - package sbrt.kernel.math.algebra Provides classes and interfaces for creating objects relevant to algebra.   sbrt.kernel.math.algebra.proc   - package sbrt.kernel.math.algebra.proc Provides classes and interfaces for executing algebra-related processes.   sbrt.kernel.math.comb.comp   - package sbrt.kernel.math.comb.comp Provides classes and interfaces for performing combinatorial analysis-related computations.   sbrt.kernel.math.comb.proc   - package sbrt.kernel.math.comb.proc Provides classes and interfaces for performing combinatorial analysis-related processes.   sbrt.kernel.math.geom   - package sbrt.kernel.math.geom Provides classes and interfaces for creating objects relevant to computational
geometry.   sbrt.kernel.math.geom.comp   - package sbrt.kernel.math.geom.comp Provides classes and interfaces for performing geometry-related computations.   sbrt.kernel.math.geom.proc   - package sbrt.kernel.math.geom.proc Provides classes and interfaces for performing geometry-related processes.   sbrt.kernel.math.graph_theory   - package sbrt.kernel.math.graph_theory Provides classes and interfaces for creating objects relevant to graph theory.   sbrt.kernel.math.graph_theory.comp   - package sbrt.kernel.math.graph_theory.comp Provides classes and interfaces for performing graph theoretical computations.   sbrt.kernel.math.graph_theory.proc   - package sbrt.kernel.math.graph_theory.proc Provides classes and interfaces for performing graph theoretical processes.   sbrt.kernel.math.optimization   - package sbrt.kernel.math.optimization Provides classes and interfaces for creating objects relevant to solving
optimization problems.   sbrt.kernel.math.stat   - package sbrt.kernel.math.stat Provides classes and interfaces for creating objects relevant to statistics.   sbrt.kernel.math.stat.comp   - package sbrt.kernel.math.stat.comp Provides classes and interfaces for performing statistical computations.   sbrt.kernel.math.stat.proc   - package sbrt.kernel.math.stat.proc Provides classes and interfaces for performing statistical processes.   sbrt.kernel.util   - package sbrt.kernel.util Provides classes and interfaces for performing utility functions.   sbrt.kernel.utilities.proc   - package sbrt.kernel.utilities.proc Provides classes and interfaces for performing utility processes.   sbrt.shell   - package sbrt.shell Provides classes used by shell-level software.   sbrt.shell.gui   - package sbrt.shell.gui Provides classes for creating graphical user interfaces for the SBRT.   sbrt.shell.io   - package sbrt.shell.io Provides classes and interfaces for reading and writing files.   sbrt.shell.io.fba   - package sbrt.shell.io.fba Provides classes and interfaces for reading and writing files relevant to
Flux Balance Analysis.   sbrt.shell.mng   - package sbrt.shell.mng Provides classes and interfaces for creating process managers.   sbrt.shell.mng.algebra   - package sbrt.shell.mng.algebra Provides classes for creating algebra process managers.   sbrt.shell.mng.comb   - package sbrt.shell.mng.comb Provides classes for creating combinatorics process managers.   sbrt.shell.mng.fba   - package sbrt.shell.mng.fba Provides classes and interfaces for creating Flux Balance Analysis
process managers.   sbrt.shell.mng.geom   - package sbrt.shell.mng.geom Provides classes and for creating geometry process managers.   sbrt.shell.mng.graph_theory   - package sbrt.shell.mng.graph_theory Provides classes and for creating graph theoretical process managers.   sbrt.shell.mng.stat   - package sbrt.shell.mng.stat Provides classes and interfaces for creating statistical
process managers.   sbrt.shell.mng.utilities   - package sbrt.shell.mng.utilities Provides classes and interfaces for creating mathematical
process managers.   sbrt.shell.proc.fba   - package sbrt.shell.proc.fba Provides classes for converting FBA-related files of different formats.   sbrt.shell.proc.graph_theory   - package sbrt.shell.proc.graph_theory     sbrt.shell.text   - package sbrt.shell.text Provides classes and interfaces for defining formats for basic objects.   sbrt.shell.text.chem   - package sbrt.shell.text.chem Provides classes and interfaces for defining formats for chemistry related 
objects.   sbrt.shell.text.fba   - package sbrt.shell.text.fba Provides classes and interfaces for defining formats for objects relevant to
Flux Balance Analysis.   sbrt.shell.text.math   - package sbrt.shell.text.math Provides classes and interfaces for defining formats for mathematical objects.   SbrtFile   - Interface in  sbrt.shell.io  This interface is used to represent files.   SbrtGui   - Class in  sbrt.shell.gui  This class is used to create a GUI for the SBRT.   SbrtGui()   - 
Constructor for class sbrt.shell.gui. SbrtGui 
 Constructs a new GUI for the SBRT.
   SEED   - 
Static variable in class sbrt.shell.mng. AbstractProcessManager 
 The keword used to denote the seed to a pseudorandom number generator.
   SEMICOLON   - 
Static variable in class sbrt.shell.text. SpecialChars 
 ;
   SenseFormat   - Class in  sbrt.shell.text.math  This class is used to represent formats of optimization senses.   SenseFormat()   - 
Constructor for class sbrt.shell.text.math. SenseFormat 
  
   SerFbaOptOutputFile   - Class in  sbrt.shell.io.fba  This class is used to write output files 
 containing FBA optimization data as serialized maps.   SerFbaOptOutputFile(String, FileFormat, Set<String>, FbaOptFileLineFormat)   - 
Constructor for class sbrt.shell.io.fba. SerFbaOptOutputFile 
 Constructs a new serialized FBA optimization output file.
   SERIALIZED   - 
Static variable in class sbrt.shell.io. FileFormat 
 Used to denote a file containing serialized objects.
   SerializedFileIterator  < E > - Class in  sbrt.shell.io  This class is used to iterate over the objects in a serialized
 input file.   SerializedFileIterator(SerializedInputFile<E>)   - 
Constructor for class sbrt.shell.io. SerializedFileIterator 
 Constructs a new serialized file iterator using the
 provided serialized input file.
   SerializedInputFile  < E > - Class in  sbrt.shell.io  This class is used to read input files containing serialized
 objects.   SerializedInputFile(String)   - 
Constructor for class sbrt.shell.io. SerializedInputFile 
 Constructs a new text input file using the provided argument.
   SerializedInputFile(String, FileFormat)   - 
Constructor for class sbrt.shell.io. SerializedInputFile 
 Constructs a new text input file using the provided arguments.
   SerializedMapInputFile  < K , V > - Class in  sbrt.shell.io  This class is used to read input files containing serialized
 maps.   SerializedMapInputFile(String, FileFormat)   - 
Constructor for class sbrt.shell.io. SerializedMapInputFile 
 Constructs a new input file using the provided arguments.
   SerializedMapInputFile(String, FileFormat, Set<? extends K>)   - 
Constructor for class sbrt.shell.io. SerializedMapInputFile 
 Constructs a new input file using the provided arguments.
   SerializedOutputFile  < E > - Class in  sbrt.shell.io  This class is used to write serialized objects to output files.   SerializedOutputFile(String, FileFormat)   - 
Constructor for class sbrt.shell.io. SerializedOutputFile 
 Constructs a new serialized output file from the provided
 arguments.
   set(int, E)   - 
Method in class sbrt.kernel.util. UnmodifiableList 
 See List.set(int, Object).
   set(File)   - 
Static method in class sbrt.shell.io. WorkingDirectory 
 Sets the working directory to the provided value.
   set(String)   - 
Static method in class sbrt.shell.io. WorkingDirectory 
 Sets the working directory to the provided value.
   setActiveFluxes(int)   - 
Method in class sbrt.kernel.fba.proc. RandomConstraintsGeneratorV1 
 Sets the total number of active fluxes to be contained in each randomly
 generated set of flux constraints.
   setActiveFluxes(int)   - 
Method in class sbrt.kernel.fba.proc. RandomEnvGeneratorV1 
 Sets the total number of active source fluxes to be contained in each
 randomly generated environment.
   setCatalystDeletionSource(Iterable<Set<String>>)   - 
Method in class sbrt.kernel.fba.proc. CvCatalystDeletionProcess 
 Sets the catalyst deletion input source.
   setCatalystDelSource(Iterable<Set<String>>)   - 
Method in class sbrt.kernel.fba.proc. CatalystDeletionProcess 
 Sets the catalyst deletion input source.
   setColExprConstraint(MathExpr<?>, Interval)   - 
Method in class sbrt.kernel.fba.comp. FbaOptComputer 
 Changes the constraint on the provided mathematical expression 
 of column variables.
   setColExprConstraints(Map<? extends MathExpr<?>, Interval>)   - 
Method in class sbrt.kernel.fba.comp. FbaOptComputer 
 Changes the constraints on more than one mathematical expression 
 of column variables.
   setColumnConstraint(T, Interval)   - 
Method in class sbrt.ext.cplex. CplexSolver 
 Sets the constraint on the provided column variable.
   setColumnConstraint(Y, Interval)   - 
Method in class sbrt.ext.glpk. GlpkSolver 
 Sets the constraint on the provided column variable.
   setColumnConstraint(Object, Interval)   - 
Method in class sbrt.kernel.fba.comp. FbaOptComputer 
 Changes the constraint of the provided column variable.
   setColumnConstraint(Y, Interval)   - 
Method in interface sbrt.kernel.math.optimization. ProgramSolver 
 Sets the constraint on the provided column variable.
   setColumnConstraints(Map<? extends C, Interval>)   - 
Method in class sbrt.ext.cplex. CplexSolver 
 Sets the constraints for the provided column variables.
   setColumnConstraints(Map<? extends C, Interval>)   - 
Method in class sbrt.ext.glpk. GlpkSolver 
 Sets the constraints for the provided column variables.
   setColumnConstraints(Map<?, Interval>)   - 
Method in class sbrt.kernel.fba.comp. FbaOptComputer 
 Changes the constraints on more than one column variable.
   setColumnConstraints(Map<? extends C, Interval>)   - 
Method in interface sbrt.kernel.math.optimization. ProgramSolver 
 Sets the constraints for the provided column variables.
   setConstraint(MathExpr<? extends C>, Interval)   - 
Method in class sbrt.ext.cplex. CplexSolver 
 Sets a constraint on the provided mathematical expression of
 column variables.
   setConstraint(LinearComb<? extends C>, Interval)   - 
Method in class sbrt.ext.cplex. CplexSolver 
 Sets a constraint on the provided mathematical expression of
 column variables.
   setConstraint(QuadraticExpr<? extends C>, Interval)   - 
Method in class sbrt.ext.cplex. CplexSolver 
 Sets a constraint on the provided mathematical expression of
 column variables.
   setConstraint(MathExpr<? extends C>, Interval)   - 
Method in class sbrt.ext.glpk. GlpkSolver 
 Sets a constraint on the provided mathematical expression of
 column variables.
   setConstraint(LinearComb<? extends C>, Interval)   - 
Method in class sbrt.ext.glpk. GlpkSolver 
 Sets a constraint on the provided mathematical expression of
 column variables.
   setConstraint(LinearComb<? extends C>, Interval)   - 
Method in interface sbrt.kernel.math.optimization. LinearProgramSolver 
 Sets a constraint on the provided linear combination of
 column variables.
   setConstraint(MathExpr<? extends C>, Interval)   - 
Method in interface sbrt.kernel.math.optimization. ProgramSolver 
 Sets a constraint on the provided mathematical expression of
 column variables.
   setConstraint(QuadraticExpr<? extends C>, Interval)   - 
Method in interface sbrt.kernel.math.optimization. QuadraticProgramSolver 
 Sets a constraint on the provided quadratic expression of
 column variables.
   setConstraints(Map<? extends MathExpr<? extends C>, Interval>)   - 
Method in class sbrt.ext.cplex. CplexSolver 
 Sets constraints on the provided mathematical expressions of
 column variables.
   setConstraints(Map<? extends MathExpr<? extends C>, Interval>)   - 
Method in class sbrt.ext.glpk. GlpkSolver 
 Sets constraints on the provided mathematical expressions of
 column variables.
   setConstraints(FluxConstraints, FluxCvComputer)   - 
Static method in class sbrt.kernel.fba.proc. CvFbaOptProcess 
 Sets the provided constraints in the provided constraint-variation 
 computer.
   setConstraints(Map<? extends MathExpr<? extends C>, Interval>)   - 
Method in interface sbrt.kernel.math.optimization. ProgramSolver 
 Sets constraints on the provided mathematical expressions of
 column variables.
   setConstraintSource(Iterable<FluxConstraints>)   - 
Method in class sbrt.kernel.fba.proc. CvCatalystDeletionProcess 
 Sets the constraint variation input source.
   setConstraintSource(Iterable<FluxConstraints>)   - 
Method in class sbrt.kernel.fba.proc. CvFbaOptimizationExperiment 
 Sets the constraint variation input source.
   setConstraintSource(Iterable<FluxConstraints>)   - 
Method in class sbrt.kernel.fba.proc. CvFluxIntervalProcess 
 Sets the constraint variation input source.
   setConstraintSource(Iterable<FluxConstraints>)   - 
Method in class sbrt.kernel.fba.proc. CvObjectiveFunctionProcess 
 Sets the constraint variation input source.
   setConstraintSource(Iterable<FluxConstraints>)   - 
Method in class sbrt.kernel.fba.proc. CvRxnDeletionProcess 
 Sets the constraint variation input source.
   setConstraintSource(Iterable<FluxConstraints>)   - 
Method in class sbrt.kernel.fba.proc. SvCdHarFluxVectorProcess 
 Sets the constraint variation input source.
   setConstraintSource(Iterable<FluxConstraints>)   - 
Method in class sbrt.kernel.fba.proc. SvInitialPointProcess 
 Sets the constraint variation input source.
   setConstraintTolerance(double)   - 
Method in class sbrt.kernel.fba.comp. CdHarFluxVectorComputer 
 Sets the constraint tolerance.
   setConstraintTolerance(double)   - 
Method in class sbrt.kernel.math.geom.comp. CdHarComputer 
 Sets the constraint tolerance.
   setConstraintTolerance(double)   - 
Method in class sbrt.kernel.math.geom.proc. CdHarProcess 
 Sets the constraint tolerance.
   setElement(X, Y, double)   - 
Method in class sbrt.kernel.math.algebra. AbstractSmdMatrix 
 Sets the element at the provided row and column indices
 to the provided double precision value.
   setElement(X, Y, double)   - 
Method in interface sbrt.kernel.math.algebra. DoubleMatrixBuilder 
 Sets the element at the provided row and column indices
 to the provided double precision value.
   setElement(X, Y, Z)   - 
Method in class sbrt.kernel.math.algebra. HashMatrix 
 Sets the element at the provided row and column indices
 to the provided double precision value.
   setElement(X, Y, Z)   - 
Method in interface sbrt.kernel.math.algebra. MatrixBuilder 
 Sets the element at the provided row and column indices
 to the one provided.
   setElement(X, Y, Z)   - 
Method in class sbrt.kernel.math.algebra. RowOrTreeMatrix 
 Sets the element at the provided row and column indices
 to the provided double precision value.
   setElement(X, Y, Z)   - 
Method in class sbrt.kernel.math.algebra. TreeMatrix 
 Sets the element at the provided row and column indices
 to the provided double precision value.
   setElements(DoubleMatrix<? extends R, ? extends C>)   - 
Method in class sbrt.kernel.math.algebra. AbstractMdMatrix 
 Sets the corresponding elements of this matrix to 
 those of the provided matrix.
   setElements(Matrix<? extends R, ? extends C, ? extends Double>)   - 
Method in class sbrt.kernel.math.algebra. AbstractSmdMatrix 
 Sets the corresponding elements of this matrix to 
 those of the provided matrix.
   setElements(SparseDoubleMatrix<? extends R, ? extends C>)   - 
Method in class sbrt.kernel.math.algebra. AbstractSmdMatrix 
 Sets the corresponding elements of this matrix to 
 those of the provided matrix.
   setElements(Map<IndexPair<R, C>, Double>)   - 
Method in class sbrt.kernel.math.algebra. AbstractSmdMatrix 
 Sets the corresponding elements of this matrix to 
 those provided.
   setElements(DoubleMatrix<? extends R, ? extends C>)   - 
Method in interface sbrt.kernel.math.algebra. DoubleMatrixBuilder 
 Sets the corresponding elements of this matrix to 
 those of the provided matrix.
   setElements(Matrix<? extends R, ? extends C, ? extends E>)   - 
Method in interface sbrt.kernel.math.algebra. MatrixBuilder 
 Sets the corresponding elements of this matrix to 
 those of the provided matrix.
   setElements(SparseDoubleMatrix<? extends R, ? extends C>)   - 
Method in interface sbrt.kernel.math.algebra. SmdMatrix 
 Sets the corresponding elements of this matrix to 
 those of the provided matrix.
   setEquations(Map<LinearComb<V>, Double>)   - 
Method in class sbrt.kernel.math.algebra.proc. LinearSolverProcess 
 Sets the system of linear equations to be solver.
   setExprs(Collection<? extends E>)   - 
Method in class sbrt.kernel.utilities.proc. VariableParticipation 
 Sets the collection of mathematical expressions to organize.
   setFluxConstraint(String, Interval)   - 
Method in class sbrt.kernel.fba.comp. AbstractFluxIntervalComputer 
 Changes the flux constraint of the reaction with the provided name.
   setFluxConstraint(String, Interval)   - 
Method in class sbrt.kernel.fba.comp. CdHarFluxVectorComputer 
 Changes the flux constraint of the reaction with the provided name.
   setFluxConstraint(String, Interval)   - 
Method in class sbrt.kernel.fba.comp. FastMsFluxRangeComputer 
 Changes the flux constraint of the reaction with the provided name.
   setFluxConstraint(String, Interval)   - 
Method in class sbrt.kernel.fba.comp. FbaOptComputer 
 Changes the flux constraint of the reaction with the provided name.
   setFluxConstraint(String, Interval)   - 
Method in interface sbrt.kernel.fba.comp. FluxCvComputer 
 Changes the flux constraint of the reaction with the provided name.
   setFluxConstraint(String, Interval)   - 
Method in class sbrt.kernel.fba.comp. InitialPointComputer 
 Changes the flux constraint of the reaction with the provided name.
   setFluxConstraint(String, Interval)   - 
Method in class sbrt.kernel.fba.comp. RxnDeletionComputer 
 Changes the flux constraint of the reaction with the provided name.
   setFluxConstraints(Map<String, Interval>)   - 
Method in class sbrt.kernel.fba.comp. AbstractCvComputer 
 Changes the flux constraints on more than one reaction.
   setFluxConstraints(Map<String, Interval>)   - 
Method in class sbrt.kernel.fba.comp. AbstractFluxIntervalComputer 
 Changes the flux constraints on more than one reaction.
   setFluxConstraints(Map<String, Interval>)   - 
Method in class sbrt.kernel.fba.comp. FastMsFluxRangeComputer 
 Changes the flux constraints on more than one reaction.
   setFluxConstraints(Map<String, Interval>)   - 
Method in class sbrt.kernel.fba.comp. FbaOptComputer 
 Changes the flux constraints on more than one reaction.
   setFluxConstraints(Map<String, Interval>)   - 
Method in interface sbrt.kernel.fba.comp. FluxCvComputer 
 Changes the flux constraints on more than one reaction.
   setFluxConstraints(Map<String, Interval>)   - 
Method in class sbrt.kernel.fba.comp. InitialPointComputer 
 Changes the flux constraints on more than one reaction.
   setFluxIntervalSource(Iterable<Map<String, Interval>>)   - 
Method in class sbrt.kernel.fba.proc. FluxPlasticityProcess 
 Sets the flux interval source.
   setFluxVectorSource(Iterable<Map<String, Double>>)   - 
Method in class sbrt.kernel.fba.proc. FluxActivityProcess 
 Sets the flux vector source.
   setFluxVectorSource(Iterable<Map<String, Double>>)   - 
Method in class sbrt.kernel.fba.proc. SvCdHarFluxVectorProcess 
 Sets the source of flux vectors to serve as initial points.
   setGraph(DirectedGraph<N>)   - 
Method in class sbrt.kernel.math.graph_theory.proc. CycleIdProcess 
 Sets the graph for which all cycles will be identified.
   setGraph(DirectedGraph<N>)   - 
Method in class sbrt.kernel.math.graph_theory.proc. PathIdProcess 
 Sets the graph for which all paths will be identified.
   setInitialFluxVector(Map<String, Double>)   - 
Method in class sbrt.kernel.fba.comp. CdHarFluxVectorComputer 
 Sets the initial flux vector to the one provided.
   setInput(ManagerInput<String, String>)   - 
Method in class sbrt.shell.mng. AbstractProcessManager 
 Uses the provided input to prepare this process manager for execution.
   setInput(ManagerInput<String, String>, PrintWriter)   - 
Method in class sbrt.shell.mng.algebra. LinearCombConverterManager 
 Uses the provided input to prepare this process manager for execution.
   setInput(ManagerInput<String, String>, PrintWriter)   - 
Method in class sbrt.shell.mng.algebra. LinearSolverManager 
 Uses the provided input to prepare this process manager for execution.
   setInput(ManagerInput<String, String>, PrintWriter)   - 
Method in class sbrt.shell.mng.algebra. MatrixConverterManager 
 Uses the provided input to prepare this process manager for execution.
   setInput(ManagerInput<String, String>, PrintWriter)   - 
Method in class sbrt.shell.mng.algebra. MultipleVectorsConverterManager 
 Uses the provided input to prepare this process manager for execution.
   setInput(ManagerInput<String, String>, PrintWriter)   - 
Method in class sbrt.shell.mng.algebra. SingleVectorConverterManager 
 Uses the provided input to prepare this process manager for execution.
   setInput(ManagerInput<String, String>, PrintWriter)   - 
Method in class sbrt.shell.mng.comb. SingleElementUnionManager 
 Uses the provided input to prepare this process manager for execution.
   setInput(ManagerInput<String, String>, PrintWriter)   - 
Method in class sbrt.shell.mng.comb. StrictSingleElementUnionManager 
 Uses the provided input to prepare this process manager for execution.
   setInput(ManagerInput<String, String>, PrintWriter)   - 
Method in class sbrt.shell.mng.fba. BiggSbmlReaderManager 
 Uses the provided input to prepare this process manager for execution.
   setInput(ManagerInput<String, String>, PrintWriter)   - 
Method in class sbrt.shell.mng.fba. BiggSbmlTranslaterManager 
 Uses the provided input to prepare this process manager for execution.
   setInput(ManagerInput<String, String>, PrintWriter)   - 
Method in class sbrt.shell.mng.fba. CatalystDeletionManager 
 Uses the provided input to prepare this process manager for execution.
   setInput(ManagerInput<String, String>, PrintWriter)   - 
Method in class sbrt.shell.mng.fba. CdHarFluxVectorManager 
 Uses the provided input to prepare this process manager for execution.
   setInput(ManagerInput<String, String>, PrintWriter)   - 
Method in class sbrt.shell.mng.fba. CvCatalystDeletionManager 
 Uses the provided input to prepare this process manager for execution.
   setInput(ManagerInput<String, String>, PrintWriter)   - 
Method in class sbrt.shell.mng.fba. CvFbaOptManager 
 Uses the provided input to prepare this process
 manager for execution.
   setInput(ManagerInput<String, String>, PrintWriter)   - 
Method in class sbrt.shell.mng.fba. CvMsFluxIntervalManager 
 Uses the provided input to prepare this process
 manager for execution.
   setInput(ManagerInput<String, String>, PrintWriter)   - 
Method in class sbrt.shell.mng.fba. CvObjectiveFunctionManager 
 Uses the provided input to prepare this process
 manager for execution.
   setInput(ManagerInput<String, String>, PrintWriter)   - 
Method in class sbrt.shell.mng.fba. CvRevRxnFluxIntervalManager 
 Uses the provided input to prepare this process manager for execution.
   setInput(ManagerInput<String, String>, PrintWriter)   - 
Method in class sbrt.shell.mng.fba. CvRxnDeletionManager 
 Uses the provided input to prepare this process
 manager for execution.
   setInput(ManagerInput<String, String>, PrintWriter)   - 
Method in class sbrt.shell.mng.fba. CvSimpleFluxIntervalManager 
 Uses the provided input to prepare this process
 manager for execution.
   setInput(ManagerInput<String, String>, PrintWriter)   - 
Method in class sbrt.shell.mng.fba. ExtremeCurrentManager 
 Uses the provided input to prepare this process
 manager for execution.
   setInput(ManagerInput<String, String>, PrintWriter)   - 
Method in class sbrt.shell.mng.fba. FbaOptManager 
 Uses the provided input to prepare this process manager for execution.
   setInput(ManagerInput<String, String>, PrintWriter)   - 
Method in class sbrt.shell.mng.fba. FbaSolverManager 
 Uses the provided input to prepare this process
 manager for execution.
   setInput(ManagerInput<String, String>, PrintWriter)   - 
Method in class sbrt.shell.mng.fba. FluxActivityManager 
 Uses the provided input to prepare this process manager for execution.
   setInput(ManagerInput<String, String>, PrintWriter)   - 
Method in class sbrt.shell.mng.fba. FluxCapManager 
 Uses the provided input to prepare this process
 manager for execution.
   setInput(ManagerInput<String, String>, PrintWriter)   - 
Method in class sbrt.shell.mng.fba. FluxPlasticityManager 
 Uses the provided input to prepare this process manager for execution.
   setInput(ManagerInput<String, String>, PrintWriter)   - 
Method in class sbrt.shell.mng.fba. InitialPointManager 
 Uses the provided input to prepare this process manager for execution.
   setInput(ManagerInput<String, String>, PrintWriter)   - 
Method in class sbrt.shell.mng.fba. MetatoolFileWriterManager 
 Uses the provided input to prepare this process manager for execution.
   setInput(ManagerInput<String, String>, PrintWriter)   - 
Method in class sbrt.shell.mng.fba. MmaNotebookManager 
 Uses the provided input to prepare this process manager for execution.
   setInput(ManagerInput<String, String>, PrintWriter)   - 
Method in class sbrt.shell.mng.fba. MsFluxIntervalManager 
 Uses the provided input to prepare this process manager for execution.
   setInput(ManagerInput<String, String>, PrintWriter)   - 
Method in class sbrt.shell.mng.fba. MsFluxomeReducerManager 
 Uses the provided input to prepare this process
 manager for execution.
   setInput(ManagerInput<String, String>, PrintWriter)   - 
Method in class sbrt.shell.mng.fba. NetworkInfoManager 
 Uses the provided input to prepare this process manager for execution.
   setInput(ManagerInput<String, String>, PrintWriter)   - 
Method in class sbrt.shell.mng.fba. ObjectiveFunctionManager 
 Uses the provided input to prepare this process
 manager for execution.
   setInput(ManagerInput<String, String>, PrintWriter)   - 
Method in class sbrt.shell.mng.fba. PalssonSbmlReaderManager 
 Uses the provided input to prepare this process manager for execution.
   setInput(ManagerInput<String, String>, PrintWriter)   - 
Method in class sbrt.shell.mng.fba. PalssonSbmlTranslaterManager 
 Uses the provided input to prepare this process manager for execution.
   setInput(ManagerInput<String, String>, PrintWriter)   - 
Method in class sbrt.shell.mng.fba. RandomConstraintsManager 
 Uses the provided input to prepare this process
 manager for execution.
   setInput(ManagerInput<String, String>, PrintWriter)   - 
Method in class sbrt.shell.mng.fba. RandomEnvManager 
 Uses the provided input to prepare this process manager for execution.
   setInput(ManagerInput<String, String>, PrintWriter)   - 
Method in class sbrt.shell.mng.fba. RandomObjectiveManager 
 Uses the provided input to prepare this process
 manager for execution.
   setInput(ManagerInput<String, String>, PrintWriter)   - 
Method in class sbrt.shell.mng.fba. ReverseRxnFluxIntervalManager 
 Uses the provided input to prepare this process manager for execution.
   setInput(ManagerInput<String, String>, PrintWriter)   - 
Method in class sbrt.shell.mng.fba. RxnDeletionManager 
 Uses the provided input to prepare this process
 manager for execution.
   setInput(ManagerInput<String, String>, PrintWriter)   - 
Method in class sbrt.shell.mng.fba. RxnRemovalManager 
 Uses the provided input to prepare this process manager for execution.
   setInput(ManagerInput<String, String>, PrintWriter)   - 
Method in class sbrt.shell.mng.fba. SbmlWriterManager 
 Uses the provided input to prepare this process manager for execution.
   setInput(ManagerInput<String, String>, PrintWriter)   - 
Method in class sbrt.shell.mng.fba. SimpleFluxIntervalManager 
 Uses the provided input to prepare this process manager for execution.
   setInput(ManagerInput<String, String>, PrintWriter)   - 
Method in class sbrt.shell.mng.fba. SimpleRxnFileTransManager 
 Uses the provided input to prepare this process manager for execution.
   setInput(ManagerInput<String, String>, PrintWriter)   - 
Method in class sbrt.shell.mng.fba. SlpCycleIdManager 
 Uses the provided input to prepare this process manager for execution.
   setInput(ManagerInput<String, String>, PrintWriter)   - 
Method in class sbrt.shell.mng.fba. StoichVariationManager 
 Uses the provided input to prepare this process manager for execution.
   setInput(ManagerInput<String, String>, PrintWriter)   - 
Method in class sbrt.shell.mng.fba. SvCdHarFluxVectorManager 
 Uses the provided input to prepare this process manager for execution.
   setInput(ManagerInput<String, String>, PrintWriter)   - 
Method in class sbrt.shell.mng.fba. SvInitialPointManager 
 Uses the provided input to prepare this process manager for execution.
   setInput(ManagerInput<String, String>, PrintWriter)   - 
Method in class sbrt.shell.mng.fba. WwFluxomeReducerManager 
 Uses the provided input to prepare this process
 manager for execution.
   setInput(ManagerInput<String, String>, PrintWriter)   - 
Method in class sbrt.shell.mng.geom. CdHarManager 
 Uses the provided input to prepare this process manager for execution.
   setInput(ManagerInput<String, String>, PrintWriter)   - 
Method in class sbrt.shell.mng.graph_theory. CycleIdManager 
 Uses the provided input to prepare this process
 manager for execution.
   setInput(ManagerInput<String, String>, PrintWriter)   - 
Method in class sbrt.shell.mng.graph_theory. PathIdManager 
 Uses the provided input to prepare this process
 manager for execution.
   setInput(ManagerInput<String, String>, PrintWriter)   - 
Method in class sbrt.shell.mng.graph_theory. UniqueCycleIdManager 
 Uses the provided input to prepare this process
 manager for execution.
   setInput(ManagerInput<K, V>)   - 
Method in interface sbrt.shell.mng. ProcessManager 
 Uses the provided input to prepare this process
 manager for execution.
   setInput(ManagerInput<K, V>, PrintWriter)   - 
Method in interface sbrt.shell.mng. ProcessManager 
 Uses the provided input to prepare this process
 manager for execution.
   setInput(ManagerInput<String, String>, PrintWriter)   - 
Method in class sbrt.shell.mng.stat. CorrelationManager 
 Uses the provided input to prepare this process manager for execution.
   setInput(ManagerInput<String, String>, PrintWriter)   - 
Method in class sbrt.shell.mng.stat. KendallTauManager 
 Uses the provided input to prepare this process
 manager for execution.
   setInput(ManagerInput<String, String>, PrintWriter)   - 
Method in class sbrt.shell.mng.stat. MannWhitneyUManager 
 Uses the provided input to prepare this process manager for
 execution.
   setInput(ManagerInput<String, String>, PrintWriter)   - 
Method in class sbrt.shell.mng.utilities. IntervalComparisonManager 
 Uses the provided input to prepare this process manager for execution.
   setInput(ManagerInput<String, String>, PrintWriter)   - 
Method in class sbrt.shell.mng.utilities. VariableParticipationManager 
 Uses the provided input to prepare this process
 manager for execution.
   setInput(ManagerInput<String, String>, PrintWriter)   - 
Method in class sbrt.shell.mng.utilities. VectorComparisonManager 
 Uses the provided input to prepare this process manager for execution.
   setIntervalSources(Iterable<Map<V, Interval>>, Iterable<Map<V, Interval>>)   - 
Method in class sbrt.kernel.utilities.proc. IntervalComparison 
  
   setIterations(int)   - 
Method in class sbrt.kernel.fba.proc. CdHarFluxVectorProcess 
 Sets the total number of flux vectors to be generated.
   setIterations(int)   - 
Method in class sbrt.kernel.fba.proc. RandomConstraintsGeneratorV1 
 Sets the number of sets of random flux constraints to be produced.
   setIterations(int)   - 
Method in class sbrt.kernel.fba.proc. RandomEnvGeneratorV1 
 Sets the number of random environments to be produced.
   setIterations(int)   - 
Method in class sbrt.kernel.fba.proc. RandomObjectiveGenerator 
 Sets the number of random objective functions to be produced.
   setIterations(int)   - 
Method in class sbrt.kernel.fba.proc. SvCdHarFluxVectorProcess 
 Sets the total number of flux vectors to be generated.
   setIterations(int)   - 
Method in class sbrt.kernel.math.geom.proc. CdHarProcess 
 Sets the total number of random points to be added to the provided
 results manager.
   setMatrixElement(S, T, double)   - 
Method in class sbrt.ext.cplex. CplexSolver 
 Sets the element of the program matrix at the provided row and 
 column variables to the one provided.
   setMatrixElement(X, Y, double)   - 
Method in class sbrt.ext.glpk. GlpkSolver 
 Sets the element of the program matrix at the provided row and 
 column variables to the one provided.
   setMatrixElement(Object, Object, double)   - 
Method in class sbrt.kernel.fba.comp. FbaOptComputer 
 Sets the specified element of the program matrix to the one provided.
   setMatrixElement(X, Y, double)   - 
Method in interface sbrt.kernel.math.optimization. ProgramSolver 
 Sets the element of the program matrix at the provided row and 
 column variables to the one provided.
   setMatrixElements(DoubleMatrix<?, ?>)   - 
Method in class sbrt.kernel.fba.comp. FbaOptComputer 
 Sets the specified elements of the program matrix to those provided.
   setMatrixSource(Iterable<Map<IndexPair<String, String>, Double>>)   - 
Method in class sbrt.kernel.fba.proc. StoichVariationProcess 
 Sets the stoiciometry variation input source.
   setMaxObstructedMoves(int)   - 
Method in class sbrt.kernel.math.geom.proc. CdHarProcess 
 Sets the maximum number of obstructed moves to allow.
   setMaxWastedMoves(int)   - 
Method in class sbrt.kernel.fba.proc. CdHarFluxVectorProcess 
 Sets the maximum number of wasted moves to allow.
   setMinChordLength(double)   - 
Method in class sbrt.kernel.fba.comp. CdHarFluxVectorComputer 
 Sets the minimum chord length.
   setMinChordLength(double)   - 
Method in class sbrt.kernel.math.geom.comp. CdHarComputer 
 Sets the minimum chord length.
   setMinChordLength(double)   - 
Method in class sbrt.kernel.math.geom.proc. CdHarProcess 
 Sets the minimum chord length.
   setObjectiveFunction(MathExpr<? extends C>, OptimizationSense)   - 
Method in class sbrt.ext.cplex. CplexSolver 
 Sets the objective function and optimization sense to the provided
 values.
   setObjectiveFunction(LinearComb<? extends C>, OptimizationSense)   - 
Method in class sbrt.ext.cplex. CplexSolver 
 Sets the objective function and optimization sense to the provided
 values.
   setObjectiveFunction(QuadraticExpr<? extends C>, OptimizationSense)   - 
Method in class sbrt.ext.cplex. CplexSolver 
 Sets the objective function and optimization sense to the provided
 values.
   setObjectiveFunction(MathExpr<? extends C>)   - 
Method in class sbrt.ext.cplex. CplexSolver 
 Sets the objective function to the provided mathematical expression of
 column variables.
   setObjectiveFunction(LinearComb<? extends C>)   - 
Method in class sbrt.ext.cplex. CplexSolver 
 Sets the objective function to the provided mathematical expression of
 column variables.
   setObjectiveFunction(QuadraticExpr<? extends C>)   - 
Method in class sbrt.ext.cplex. CplexSolver 
 Not yet implemented.
   setObjectiveFunction(MathExpr<? extends C>, OptimizationSense)   - 
Method in class sbrt.ext.glpk. GlpkSolver 
 Sets the objective function and optimization sense to the provided
 values.
   setObjectiveFunction(MathExpr<? extends C>)   - 
Method in class sbrt.ext.glpk. GlpkSolver 
 Sets the objective function to the provided mathematical expression of
 column variables.
   setObjectiveFunction(LinearComb<? extends C>, OptimizationSense)   - 
Method in class sbrt.ext.glpk. GlpkSolver 
 Sets the objective function and optimization sense to the provided
 values.
   setObjectiveFunction(LinearComb<? extends C>)   - 
Method in class sbrt.ext.glpk. GlpkSolver 
 Sets the objective function to the provided mathematical expression of
 column variables.
   setObjectiveFunction(MathExpr<String>)   - 
Method in class sbrt.kernel.fba.comp. RxnDeletionComputer 
 Sets the objective function to the provided mathematical expression.
   setObjectiveFunction(MathExpr<String>)   - 
Method in class sbrt.kernel.fba.proc. CatalystDeletionProcess 
 Sets the objective function to the provided mathematical expression.
   setObjectiveFunction(MathExpr<String>)   - 
Method in class sbrt.kernel.fba.proc. CvCatalystDeletionProcess 
 Sets the objective function to the provided mathematical expression.
   setObjectiveFunction(MathExpr<String>)   - 
Method in class sbrt.kernel.fba.proc. CvFbaOptimizationExperiment 
 Sets the objective function to the provided mathematical expression.
   setObjectiveFunction(MathExpr<String>)   - 
Method in class sbrt.kernel.fba.proc. CvRxnDeletionProcess 
 Sets the objective function to the provided mathematical expression.
   setObjectiveFunction(LinearComb<String>)   - 
Method in class sbrt.kernel.fba.proc. FbaOptimizationProcess 
 Sets the objective function to the provided mathematical expression.
   setObjectiveFunction(MathExpr<String>)   - 
Method in class sbrt.kernel.fba.proc. RxnDeletionProcess 
 Sets the objective function to the provided mathematical expression.
   setObjectiveFunction(MathExpr<String>)   - 
Method in class sbrt.kernel.fba.proc. StoichVariationProcess 
 Sets the objective function to the provided mathematical expression.
   setObjectiveFunction(LinearComb<? extends C>, OptimizationSense)   - 
Method in interface sbrt.kernel.math.optimization. LinearProgramSolver 
 Sets the objective function and optimization sense to the provided
 values.
   setObjectiveFunction(LinearComb<? extends C>)   - 
Method in interface sbrt.kernel.math.optimization. LinearProgramSolver 
 Sets the objective function to the provided linear combination of
 column variables.
   setObjectiveFunction(MathExpr<? extends C>, OptimizationSense)   - 
Method in interface sbrt.kernel.math.optimization. ProgramSolver 
 Sets the objective function and optimization sense to the provided
 values.
   setObjectiveFunction(MathExpr<? extends C>)   - 
Method in interface sbrt.kernel.math.optimization. ProgramSolver 
 Sets the objective function to the provided mathematical expression of
 column variables.
   setObjectiveFunction(QuadraticExpr<? extends C>, OptimizationSense)   - 
Method in interface sbrt.kernel.math.optimization. QuadraticProgramSolver 
 Sets the objective function and optimization sense to the provided
 values.
   setObjectiveFunction(QuadraticExpr<? extends C>)   - 
Method in interface sbrt.kernel.math.optimization. QuadraticProgramSolver 
 Sets the objective function to the provided quadratic expression of
 column variables.
   setObjectiveFunctionSource(Iterable<? extends MathExpr<String>>)   - 
Method in class sbrt.kernel.fba.proc. CvObjectiveFunctionProcess 
 Sets the objective function input source.
   setObjectiveFunctionSource(Iterable<? extends MathExpr<String>>)   - 
Method in class sbrt.kernel.fba.proc. ObjectiveFunctionAnalysis 
 Sets the objective function input source.
   setOff(Interval)   - 
Method in class sbrt.kernel.fba.proc. RandomConstraintsGeneratorV1 
 Sets the constraint used to represent an inactive flux.
   setOff(Interval)   - 
Method in class sbrt.kernel.fba.proc. RandomEnvGeneratorV1 
 Sets the constraint used to represent an inactive source flux.
   setOn(Interval)   - 
Method in class sbrt.kernel.fba.proc. RandomConstraintsGeneratorV1 
 Sets the constraint used to represent an active flux.
   setOn(Interval)   - 
Method in class sbrt.kernel.fba.proc. RandomEnvGeneratorV1 
 Sets the constraint used to represent an active source flux.
   setOptimizationSense(OptimizationSense)   - 
Method in class sbrt.ext.cplex. CplexSolver 
 Sets the optimization sense.
   setOptimizationSense(OptimizationSense)   - 
Method in class sbrt.ext.glpk. GlpkSolver 
 Sets the optimization sense.
   setOptimizationSense(OptimizationSense)   - 
Method in class sbrt.kernel.fba.comp. RxnDeletionComputer 
 Sets the optimization sense to the provided value.
   setOptimizationSense(OptimizationSense)   - 
Method in class sbrt.kernel.fba.proc. CatalystDeletionProcess 
 Sets the optimization sense to the provided value.
   setOptimizationSense(OptimizationSense)   - 
Method in class sbrt.kernel.fba.proc. CvCatalystDeletionProcess 
 Sets the optimization sense to the provided value.
   setOptimizationSense(OptimizationSense)   - 
Method in class sbrt.kernel.fba.proc. CvFbaOptimizationExperiment 
 Sets the optimization sense to the provided value.
   setOptimizationSense(OptimizationSense)   - 
Method in class sbrt.kernel.fba.proc. CvObjectiveFunctionProcess 
 Sets the optimization sense to the provided value.
   setOptimizationSense(OptimizationSense)   - 
Method in class sbrt.kernel.fba.proc. CvRxnDeletionProcess 
 Sets the optimization sense to the provided value.
   setOptimizationSense(OptimizationSense)   - 
Method in class sbrt.kernel.fba.proc. FbaOptimizationProcess 
 Sets the optimization sense to the provided value.
   setOptimizationSense(OptimizationSense)   - 
Method in class sbrt.kernel.fba.proc. ObjectiveFunctionAnalysis 
 Sets the optimization sense to the provided value.
   setOptimizationSense(OptimizationSense)   - 
Method in class sbrt.kernel.fba.proc. RxnDeletionProcess 
 Sets the optimization sense to the provided value.
   setOptimizationSense(OptimizationSense)   - 
Method in class sbrt.kernel.fba.proc. StoichVariationProcess 
 Sets the optimization sense to the provided value.
   setOptimizationSense(OptimizationSense)   - 
Method in interface sbrt.kernel.math.optimization. ProgramSolver 
 Sets the optimization sense.
   setParamFile(String)   - 
Method in class sbrt.ext.cplex. CplexSolver 
 Reads the program solver parameter file with the provided name.
   setParamFile(String)   - 
Method in class sbrt.ext.glpk. GlpkSolver 
 Throws  UnsupportedOperationException .
   setParamFile(String)   - 
Method in interface sbrt.kernel.math.optimization. ProgramSolver 
 Reads the program solver parameter file with the provided name.
   setPIntercept(double)   - 
Method in class sbrt.kernel.fba.comp. InitialPointComputer 
 Sets the value of the positioning variables at the edges of the 
 convex polytope.
   setPoint(Map<? extends V, Double>)   - 
Method in class sbrt.kernel.math.geom.comp. CdHarComputer 
 Sets the initial point.
   setPoint(Map<? extends V, Double>)   - 
Method in class sbrt.kernel.math.geom.proc. CdHarProcess 
 Sets the initial point.
   setPointsPerPoint(int)   - 
Method in class sbrt.kernel.fba.comp. CdHarFluxVectorComputer 
 Sets the number of points to generate for each point added to the 
 specified results manager.
   setPointsPerPoint(int)   - 
Method in class sbrt.kernel.fba.proc. CdHarFluxVectorProcess 
 Sets the number of points to generate for each point added to the 
 specified results manager.
   setPointsPerPoint(int)   - 
Method in class sbrt.kernel.math.geom.proc. CdHarProcess 
 Sets the total number of points to generate for each point added to the
 provided results manager.
   setRandom(Random)   - 
Method in class sbrt.kernel.fba.comp. CdHarFluxVectorComputer 
 Sets the random number generator.
   setRandom(Random)   - 
Method in class sbrt.kernel.fba.proc. RandomConstraintsGeneratorV1 
 Sets the random number generator to the one provided.
   setRandom(Random)   - 
Method in class sbrt.kernel.fba.proc. RandomEnvGeneratorV1 
 Sets the random number generator to the one provided.
   setRandom(Random)   - 
Method in class sbrt.kernel.fba.proc. RandomObjectiveGenerator 
 Sets the random number generator to the one provided.
   setRandom(Random)   - 
Method in class sbrt.kernel.math.geom.comp. CdHarComputer 
 Sets the random number generator.
   setRandom(Random)   - 
Method in class sbrt.kernel.math.geom.proc. CdHarProcess 
 Sets the random number generator.
   setReactionNames(Collection<String>)   - 
Method in class sbrt.kernel.fba.proc. CvFluxIntervalProcess 
 Sets the names of reactions for which flux intervals are to be computed.
   setReactionNames(Set<String>)   - 
Method in class sbrt.kernel.fba.proc. FluxIntervalProcess 
 Sets the names of reactions for which flux ranges are to be computed.
   setReactions(Set<String>)   - 
Method in class sbrt.kernel.fba.proc. RandomConstraintsGeneratorV1 
 Sets the reaction names for which random constraints are to be generated.
   setRowConstraint(T, Interval)   - 
Method in class sbrt.ext.cplex. CplexSolver 
 Sets the constraint for the provided row variable.
   setRowConstraint(X, Interval)   - 
Method in class sbrt.ext.glpk. GlpkSolver 
 Sets the constraint for the provided row variable.
   setRowConstraint(Object, Interval)   - 
Method in class sbrt.kernel.fba.comp. FbaOptComputer 
 Changes the constraint of the provided row variable.
   setRowConstraint(X, Interval)   - 
Method in interface sbrt.kernel.math.optimization. ProgramSolver 
 Sets the constraint for the provided row variable.
   setRowConstraints(Map<? extends R, Interval>)   - 
Method in class sbrt.ext.cplex. CplexSolver 
 Sets the constraints for the provided row variables.
   setRowConstraints(Map<? extends R, Interval>)   - 
Method in class sbrt.ext.glpk. GlpkSolver 
 Sets the constraints for the provided row variables.
   setRowConstraints(Map<?, Interval>)   - 
Method in class sbrt.kernel.fba.comp. FbaOptComputer 
 Changes the constraints on more than one row variable.
   setRowConstraints(Map<? extends R, Interval>)   - 
Method in interface sbrt.kernel.math.optimization. ProgramSolver 
 Sets the constraints for the provided row variables.
   setRxnDeletionSource(Iterable<Set<String>>)   - 
Method in class sbrt.kernel.fba.proc. CvRxnDeletionProcess 
 Sets the reaction deletion input source.
   setRxnDeletionSource(Iterable<Set<String>>)   - 
Method in class sbrt.kernel.fba.proc. RxnDeletionProcess 
 Sets the reaction deletion input source.
   setRxnExprConstraint(MathExpr<String>, Interval)   - 
Method in class sbrt.kernel.fba.comp. AbstractFluxIntervalComputer 
 Changes the constraint on the provided mathematical expression 
 of fluxes.
   setRxnExprConstraint(MathExpr<String>, Interval)   - 
Method in class sbrt.kernel.fba.comp. CdHarFluxVectorComputer 
 Changes the constraint on the provided mathematical expression of fluxes.
   setRxnExprConstraint(LinearComb<String>, Interval)   - 
Method in class sbrt.kernel.fba.comp. CdHarFluxVectorComputer 
 Changes the constraint on the provided linear combination of fluxes.
   setRxnExprConstraint(MathExpr<String>, Interval)   - 
Method in class sbrt.kernel.fba.comp. FastMsFluxRangeComputer 
 Changes the constraint on the provided mathematical expression 
 of fluxes.
   setRxnExprConstraint(MathExpr<String>, Interval)   - 
Method in class sbrt.kernel.fba.comp. FbaOptComputer 
 Changes the constraint on the provided mathematical expression 
 of fluxes.
   setRxnExprConstraint(MathExpr<String>, Interval)   - 
Method in interface sbrt.kernel.fba.comp. FluxCvComputer 
 Changes the constraint on the provided mathematical expression 
 of fluxes.
   setRxnExprConstraint(MathExpr<String>, Interval)   - 
Method in class sbrt.kernel.fba.comp. InitialPointComputer 
 Changes the constraint on the provided mathematical expression 
 of fluxes.
   setRxnExprConstraint(MathExpr<String>, Interval)   - 
Method in class sbrt.kernel.fba.comp. RxnDeletionComputer 
 Changes the constraint on the provided mathematical expression 
 of fluxes.
   setRxnExprConstraints(Map<? extends MathExpr<String>, Interval>)   - 
Method in class sbrt.kernel.fba.comp. AbstractCvComputer 
 Changes the constraints on more than one mathematical expression 
 of fluxes.
   setRxnExprConstraints(Map<? extends MathExpr<String>, Interval>)   - 
Method in class sbrt.kernel.fba.comp. AbstractFluxIntervalComputer 
 Changes the constraints on more than one mathematical expression 
 of fluxes.
   setRxnExprConstraints(Map<? extends MathExpr<String>, Interval>)   - 
Method in class sbrt.kernel.fba.comp. FastMsFluxRangeComputer 
 Changes the constraints on more than one mathematical expression 
 of fluxes.
   setRxnExprConstraints(Map<? extends MathExpr<String>, Interval>)   - 
Method in class sbrt.kernel.fba.comp. FbaOptComputer 
 Changes the constraints on more than one mathematical expression 
 of fluxes.
   setRxnExprConstraints(Map<? extends MathExpr<String>, Interval>)   - 
Method in interface sbrt.kernel.fba.comp. FluxCvComputer 
 Changes the constraints on more than one mathematical expression 
 of fluxes.
   setRxnExprConstraints(Map<? extends MathExpr<String>, Interval>)   - 
Method in class sbrt.kernel.fba.comp. InitialPointComputer 
 Changes the constraints on more than one mathematical expression 
 of fluxes.
   SETS_FILE   - 
Static variable in class sbrt.shell.mng.comb. SingleElementUnionManager 
 The keyword used to denote the name of the file containing the collection
 of sets.
   setSafetyLevel(int)   - 
Method in class sbrt.kernel.fba.comp. FbaOptComputer 
 Sets the safety level.
   setSampleA(List<? extends Number>)   - 
Method in class sbrt.kernel.math.stat.proc. MwUAnalysis 
 Sets sample A to the provided list of numbers.
   setSampleB(List<? extends Number>)   - 
Method in class sbrt.kernel.math.stat.proc. MwUAnalysis 
 Sets sample B to the provided list of numbers.
   setSets(Collection<Set<E>>)   - 
Method in class sbrt.kernel.math.comb.proc. SingleElementUnionProc 
 Sets the collection of sets to that provided.
   setSets(Collection<Set<E>>)   - 
Method in class sbrt.kernel.math.comb.proc. StrictSingleElementUnionProc 
 Sets the collection of sets to that provided.
   setSolver(LinearSystemSolver<String>)   - 
Method in class sbrt.kernel.fba.proc. FbaSolverProcess 
 Sets the linear system solver to be used.
   setSolver(LinearSystemSolver<V>)   - 
Method in class sbrt.kernel.math.algebra.proc. LinearSolverProcess 
 Sets the linear system solver.
   setTolerance(double)   - 
Method in class sbrt.kernel.fba.comp. FbaOptComputer 
 Sets the constraint tolerance.
   setTolerance(double)   - 
Method in class sbrt.kernel.utilities.proc. IntervalComparison 
  
   setTolerance(double)   - 
Method in class sbrt.kernel.utilities.proc. VectorComparison 
  
   setTotal(int)   - 
Method in class sbrt.kernel.util. PercentProgressMonitor 
 Sets the total number of times  increment()  can be called.
   setVectorSource(Iterator<Map<K, V>>)   - 
Method in class sbrt.kernel.math.algebra.proc. VectorIntersection 
 Sets the vector source.
   setVectorSource(Iterable<Map<V, Double>>)   - 
Method in class sbrt.kernel.utilities.proc. VectorComparison 
  
   setXValues(Map<? extends V, Double>)   - 
Method in class sbrt.kernel.math.stat.proc. CorrelationAnalysis 
 Sets the x values.
   setXValues(Map<? extends V, Double>)   - 
Method in class sbrt.kernel.math.stat.proc. MultipleCorrelationAnalysis 
 Sets the x values.
   setYValues(Map<? extends V, Double>)   - 
Method in class sbrt.kernel.math.stat.proc. CorrelationAnalysis 
 Sets the y values.
   setYValues(Iterable<Map<V, Double>>)   - 
Method in class sbrt.kernel.math.stat.proc. MultipleCorrelationAnalysis 
 Sets the source of y values.
   setZeroCutoff(double)   - 
Method in class sbrt.kernel.fba.comp. RxnDeletionComputer 
 Sets the zero cutoff to the provided value.
   setZeroCutoff(double)   - 
Method in class sbrt.kernel.fba.proc. CatalystDeletionProcess 
 Sets the zero cutoff to the provided value.
   setZeroCutoff(double)   - 
Method in class sbrt.kernel.fba.proc. CvCatalystDeletionProcess 
 Sets the zero cutoff to the provided value.
   setZeroCutoff(double)   - 
Method in class sbrt.kernel.fba.proc. CvRxnDeletionProcess 
 Sets the zero cutoff to the provided value.
   setZeroCutoff(double)   - 
Method in class sbrt.kernel.fba.proc. FluxActivityProcess 
 Sets the zero cutoff to the provided value.
   setZeroCutoff(double)   - 
Method in class sbrt.kernel.fba.proc. FluxPlasticityProcess 
 Sets the zero cutoff to the provided value.
   setZeroCutoff(double)   - 
Method in class sbrt.kernel.fba.proc. RxnDeletionProcess 
 Sets the zero cutoff to the provided value.
   SfoofSource   - Class in  sbrt.shell.io.fba  This class is used to provide a source of serialized
 FBA optimization output files.   SfoofSource(Iterator<String>, FileFormat, Set<String>, FbaOptFileLineFormat)   - 
Constructor for class sbrt.shell.io.fba. SfoofSource 
 Constructs a new source of serialized
 FBA optimization output files.
   SfoofSource(Iterator<String>, String, FileFormat, Set<String>, FbaOptFileLineFormat)   - 
Constructor for class sbrt.shell.io.fba. SfoofSource 
 Constructs a new source of serialized
 FBA optimization output files.
   ShellProcessManager  < K , V > - Interface in  sbrt.shell.mng  This interface is used to represent shell process managers.   SimpleFluxIntervalComputer   - Class in  sbrt.kernel.fba.comp  This class is used to compute flux intervals in the simplest possible way.   SimpleFluxIntervalComputer(FbaOptComputer, double)   - 
Constructor for class sbrt.kernel.fba.comp. SimpleFluxIntervalComputer 
 Constructs a new flux interval computer.
   SimpleFluxIntervalManager   - Class in  sbrt.shell.mng.fba  This class is used to manage simple flux interval experiments.   SimpleFluxIntervalManager()   - 
Constructor for class sbrt.shell.mng.fba. SimpleFluxIntervalManager 
  
   SimpleFormat  < T > - Interface in  sbrt.shell.text  This interface is used to represent simple formats.   SimpleListFormat  < E > - Class in  sbrt.shell.text  This interface is used to wrap  ListFormat  objects as  SimpleFormat 
 objects.   SimpleListFormat(ListFormat<E>)   - 
Constructor for class sbrt.shell.text. SimpleListFormat 
 Constructs a new simple list format from the provided list format.
   SimpleRxnFileTranslator   - Class in  sbrt.shell.proc.fba  This class is used to translate simple reaction files from, say,
 spreadsheets into FBA Reaction Files.   SimpleRxnFileTranslator()   - 
Constructor for class sbrt.shell.proc.fba. SimpleRxnFileTranslator 
 Constructs a new translator.
   SimpleRxnFileTransManager   - Class in  sbrt.shell.mng.fba  This class is used to manage the translation of simple reaction files files
 into Systems Biology Research Tool (SBRT) FBA Reaction Files.   SimpleRxnFileTransManager()   - 
Constructor for class sbrt.shell.mng.fba. SimpleRxnFileTransManager 
  
   SimpleSetFormat  < E > - Class in  sbrt.shell.text  This interface is used to wrap  ListFormat  objects as  SimpleFormat 
 objects.   SimpleSetFormat(LinkedHashSetFormat<E>)   - 
Constructor for class sbrt.shell.text. SimpleSetFormat 
 Constructs a new simple set format from the provided set format.
   simplify(LinearSystemSolution<V>, Map<? extends V, Interval>, double)   - 
Static method in class sbrt.kernel.math.algebra. HashLinearSystemSolution 
 Returns a simplified version of the provided linear system solution
 given the variable bounds.
   simplify(Map<?, Double>, double)   - 
Method in class sbrt.kernel.math.algebra. HashLinearSystemSolution 
 Computes and returns a simplified solution by plugging in the provided
 values.
   simplify(Map<?, Double>, double)   - 
Method in interface sbrt.kernel.math.algebra. LinearSystemSolution 
 Computes and returns a simplified solution by plugging in the provided
 values.
   SingleElementUnionManager   - Class in  sbrt.shell.mng.comb  This class is used to manage single-element union computers.   SingleElementUnionManager()   - 
Constructor for class sbrt.shell.mng.comb. SingleElementUnionManager 
  
   SingleElementUnionProc  < E > - Class in  sbrt.kernel.math.comb.proc  This class is used to wrap the single element union computer as a process.   SingleElementUnionProc()   - 
Constructor for class sbrt.kernel.math.comb.proc. SingleElementUnionProc 
 Constructs a new single-element union process.
   SingleElementUnionsV1   - Class in  sbrt.kernel.math.comb.comp  This class is used to compute the single-element unions of collections of 
 sets.   SingleElementUnionsV1()   - 
Constructor for class sbrt.kernel.math.comb.comp. SingleElementUnionsV1 
  
   SingleElementUnionsV2   - Class in  sbrt.kernel.math.comb.comp  This class is used to compute the single-element unions of collections of 
 sets.   SingleElementUnionsV2()   - 
Constructor for class sbrt.kernel.math.comb.comp. SingleElementUnionsV2 
  
   SingleRm  < R > - Class in  sbrt.kernel.io  This class is used to store a single result that can be easily retrieved.   SingleRm()   - 
Constructor for class sbrt.kernel.io. SingleRm 
  
   singleTerm(V, double)   - 
Static method in class sbrt.kernel.math. HashLinearComb 
 Returns a single-term linear combination with the provided variable and
 coefficient.
   singleTerm(V, double, double)   - 
Static method in class sbrt.kernel.math. HashLinearComb 
 Returns a single-term linear combination with the provided variable,
 coefficient, and zero cutoff.
   singleTerm(double, V)   - 
Static method in class sbrt.kernel.math. HashLinearComb 
 Returns a single-term linear combination with the provided variable and
 coefficient.
   singleTerm(V)   - 
Static method in class sbrt.kernel.math. HashLinearComb 
 Returns a single-term linear combination with the provided variable.
   singleTerm(LinearComb.Term<V>)   - 
Static method in class sbrt.kernel.math. HashLinearComb 
 Returns a single-term linear combination with the provided term.
   SingleVectorConverterManager   - Class in  sbrt.shell.mng.algebra  This class is used to convert multiple files containing single vectors into a single 
 file containing multiple vectors.   SingleVectorConverterManager()   - 
Constructor for class sbrt.shell.mng.algebra. SingleVectorConverterManager 
  
   SinkRxn   - Class in  sbrt.kernel.fba  This class is used to represent sink reactions in Flux Balance Analysis.   SinkRxn(String)   - 
Constructor for class sbrt.kernel.fba. SinkRxn 
 Constructs a new sink reaction for the provided chemical species.
   sinkRxns()   - 
Method in interface sbrt.kernel.fba. IrrevRxnFluxome 
 Returns the total number of sink reactions contained 
 in this fluxome.
   sinkRxns()   - 
Method in class sbrt.kernel.fba. IrrevRxnFluxomeV1 
 Returns the total number of sink reactions contained 
 in this fluxome.
   size()   - 
Method in class sbrt.kernel.util. UnmodifiableCollection 
 See Collection.size().
   size()   - 
Method in class sbrt.kernel.util. UnmodifiableList 
 See List.size().
   size()   - 
Method in class sbrt.kernel.util. UnmodifiableMap 
 See Map.size().
   size()   - 
Method in class sbrt.kernel.util. UnmodifiableSet 
 See Set.size().
   SlpCycleComputer   - Class in  sbrt.kernel.fba.comp  This class is used to identify the steady state cycles (Type III extreme
 pathways) in a fluxome.   SlpCycleComputer(ExtremeCurrentComputer)   - 
Constructor for class sbrt.kernel.fba.comp. SlpCycleComputer 
 Constructs a new steady state cycle computer from the given extreme
 current computer.
   SlpCycleIdManager   - Class in  sbrt.shell.mng.fba  This class is used to manage steady state cycle identification.   SlpCycleIdManager()   - 
Constructor for class sbrt.shell.mng.fba. SlpCycleIdManager 
  
   SlpExCurrComputerV2   - Class in  sbrt.kernel.fba.comp  This class is an implementation of an extreme current algorithm.   SlpExCurrComputerV2()   - 
Constructor for class sbrt.kernel.fba.comp. SlpExCurrComputerV2 
 Constructs a new extreme current computer.
   SmdMatrix  < R , C > - Interface in  sbrt.kernel.math.algebra  This interface is used to represent sparse mutable matrices 
 containing strictly double precision numbers as elements.   SmoFile  < K , V > - Class in  sbrt.shell.io  This class is used to write serialized maps to output files.   SmoFile(String, FileFormat, Set<? extends K>)   - 
Constructor for class sbrt.shell.io. SmoFile 
 Constructs a new serialized map output file from the
 provided arguments.
   SmoFileSource  < K , V > - Class in  sbrt.shell.io  This class is used to provided a source of serialized map output files.   SmoFileSource(Iterator<String>, FileFormat, Set<? extends K>)   - 
Constructor for class sbrt.shell.io. SmoFileSource 
 Constructs a new source of serialized map output files.
   SOLUTION_FILE_NAME   - 
Static variable in class sbrt.shell.mng.fba. MmaNotebookManager 
 The keyword used to denote the name of the file written by the
 Mathematica notebook.
   solve(Map<LinearComb<V>, Double>)   - 
Method in class sbrt.ext.mma.algebra. MathematicaSolver 
 Computes and returns a solution to the provided system of linear
 equations.
   solve(Fluxome, LinearSystemSolver<String>)   - 
Static method in class sbrt.kernel.fba.comp. FbaSolver 
 Solves  Sv = 0 , where  S  is the stoichiometry matrix of the provided
 fluxome and  v  is the vector of fluxes.
   solve(Map<LinearComb<V>, Double>)   - 
Method in interface sbrt.kernel.math.algebra. LinearSystemSolver 
 Computes and returns a solution to the provided system of linear equations.
   solveFor(Object, double)   - 
Method in class sbrt.kernel.math. HashLinearComb 
 Equates this linear combination to the provided constant and solves for
 the provided variable.
   solveFor(Object, double)   - 
Method in interface sbrt.kernel.math. LinearComb 
 Equates this linear combination to the provided constant and solves for
 the provided variable.
   SOLVER   - 
Static variable in class sbrt.shell. ProgramSolverFactory 
 The keyword used to denote the name of a program solver.
   SourceRxn   - Class in  sbrt.kernel.fba  This class is used to represent chemical species sources in Flux Balance
 Analysis.   SourceRxn(String)   - 
Constructor for class sbrt.kernel.fba. SourceRxn 
 Constructs a new source for the provided chemical species.
   sourceRxns()   - 
Method in interface sbrt.kernel.fba. IrrevRxnFluxome 
 Returns the total number of source reactions contained 
 in this fluxome.
   sourceRxns()   - 
Method in class sbrt.kernel.fba. IrrevRxnFluxomeV1 
 Returns the total number of source reactions contained 
 in this fluxome.
   SparseDoubleMatrix  < R , C > - Interface in  sbrt.kernel.math.algebra  This interface is used to represent sparse matrices whose
 elements are strictly double precision numbers.   SparseDoubleMatrixWrap  < R , C > - Class in  sbrt.kernel.math.algebra  This class is used to wrap mutable matrices as an immutable
  SparseDoubleMatrix .   SparseDoubleMatrixWrap(SparseDoubleMatrix<R, C>)   - 
Constructor for class sbrt.kernel.math.algebra. SparseDoubleMatrixWrap 
 Constructs a new wrapper around the provided matrix.
   SPEARMANS_RHO   - 
Static variable in class sbrt.shell.mng.stat. CorrelationManager 
 The value used to denote a Spearman's rho correlation coefficient.
   SpecialChars   - Class in  sbrt.shell.text  This class contains a set of characters that could be considered as  special 
 in some situations.   species()   - 
Method in interface sbrt.kernel.fba. Fluxome 
 Returns the total number of chemical species contained in this fluxome.
   species()   - 
Method in class sbrt.kernel.fba. IrrevRxnFluxomeV1 
 Returns the total number of chemical species contained in this fluxome.
   start()   - 
Method in class sbrt.kernel.util. AbstractProgressMonitor 
 Causes this progress monitor to start monitoring a duration of time.
   start()   - 
Method in interface sbrt.kernel.util. MutableProgressMonitor 
 Causes this progress monitor to start monitoring a duration of time.
   started()   - 
Method in class sbrt.kernel.util. AbstractProgressMonitor 
 Indicates if this progress monitor has already been started.
   started()   - 
Method in interface sbrt.kernel.util. ProgressMonitor 
 Indicates if this progress monitor has already been started.
   startR()   - 
Static method in class sbrt.ext.R. R 
 Starts the R engine if it is not already running.
   startR(PrintWriter)   - 
Static method in class sbrt.shell.mng.stat. CorrelationManager 
 Starts the R engine and writes an informative message to the provided
 print writer.
   StoichVariationManager   - Class in  sbrt.shell.mng.fba  This class is used to manage stoichiometry variation-FBA optimization
 processes.   StoichVariationManager()   - 
Constructor for class sbrt.shell.mng.fba. StoichVariationManager 
  
   StoichVariationProcess   - Class in  sbrt.kernel.fba.proc  This class is used to perform stoichiometry-variation FBA optimizations.   StoichVariationProcess(FbaOptComputer)   - 
Constructor for class sbrt.kernel.fba.proc. StoichVariationProcess 
 Constructs a new constraint-variation FBA optimization process.
   stop()   - 
Method in class sbrt.kernel.util. AbstractProgressMonitor 
 Causes this progress monitor to stop monitoring a duration of time.
   stop()   - 
Method in interface sbrt.kernel.util. MutableProgressMonitor 
 Causes this progress monitor to stop monitoring a duration of time.
   stopped()   - 
Method in class sbrt.kernel.util. AbstractProgressMonitor 
 Indicates if this progress monitor has already been stopped.
   stopped()   - 
Method in interface sbrt.kernel.util. ProgressMonitor 
 Indicates if this progress monitor has already been stopped.
   StrictSingleElementUnionManager   - Class in  sbrt.shell.mng.comb  This class is used to manage single-element union computers.   StrictSingleElementUnionManager()   - 
Constructor for class sbrt.shell.mng.comb. StrictSingleElementUnionManager 
  
   StrictSingleElementUnionProc  < E > - Class in  sbrt.kernel.math.comb.proc  This class is used to wrap the strict single-element union computer as a process.   StrictSingleElementUnionProc()   - 
Constructor for class sbrt.kernel.math.comb.proc. StrictSingleElementUnionProc 
 Constructs a new strict single-element union process.
   StrictSingleElementUnions   - Class in  sbrt.kernel.math.comb.comp  This class is used to compute the single-element unions of collections of
 sets.   StrictSingleElementUnions()   - 
Constructor for class sbrt.kernel.math.comb.comp. StrictSingleElementUnions 
 Constructs a new strict single-element union computer.
   StringFormat   - Class in  sbrt.shell.text  This class is used to define a format for  String  
 objects.   StringFormat()   - 
Constructor for class sbrt.shell.text. StringFormat 
  
   subList(int, int)   - 
Method in class sbrt.kernel.util. UnmodifiableList 
 See List.subList(int, int).
   successors(Object)   - 
Method in class sbrt.kernel.math.graph_theory. DirectedGraph 
 Returns the number of successors of the provided node.
   SURR   - 
Static variable in class sbrt.shell.text.fba. IrrevRxnFormatV1 
 The string used to denote the  surroundings .
   SvCdHarFluxVectorManager   - Class in  sbrt.shell.mng.fba  This class is used to manage flux space variation, coordinate direction
 hit-and-run random flux vector experiments.   SvCdHarFluxVectorManager()   - 
Constructor for class sbrt.shell.mng.fba. SvCdHarFluxVectorManager 
  
   SvCdHarFluxVectorProcess   - Class in  sbrt.kernel.fba.proc  This class is used to perform space variation coordinate direction 
 hit-and-run flux vector experiments.   SvCdHarFluxVectorProcess(CdHarFluxVectorComputer)   - 
Constructor for class sbrt.kernel.fba.proc. SvCdHarFluxVectorProcess 
 Constructs a new constraint variation coordinate direction hit-and-run 
 flux vector experiment.
   SvInitialPointManager   - Class in  sbrt.shell.mng.fba  This class is used to manage flux space variation-initial point experiments.   SvInitialPointManager()   - 
Constructor for class sbrt.shell.mng.fba. SvInitialPointManager 
  
   SvInitialPointProcess   - Class in  sbrt.kernel.fba.proc  This class is used to perform constraint variation initial point experiments.   SvInitialPointProcess(InitialPointComputer)   - 
Constructor for class sbrt.kernel.fba.proc. SvInitialPointProcess 
 Constructs a new constraint variation initial point experiment.
   SYSTEM_SOLUTION_FILE   - 
Static variable in class sbrt.shell.mng.fba. FbaManager 
 The keyword used to denote a file containing a solution to the system
 of linear equations formed by a fluxome.
 
 


 
   
  
 
 
 
   
 
   
          Overview     
        Package   
        Class   
        Use   
          Tree     
          Deprecated     
       Index    
          Help     
   
 
 
  
 
 
 

 
  
   PREV LETTER   
   NEXT LETTER    
  
    FRAMES     
   NO FRAMES     
  
    All Classes  ');
  }
  //-->
 
 
    All Classes  
 


  
 
 
  
 

 A   B   C   D   E   F   G   H   I   J   K   L   M   N   O   P   Q   R   S   T   U   V   W   X   Y   Z
